# Enhancing Applicability of Reversible UV Thermochromic Offset Inks: Edge Quality Parameters and Thermochromic Printing System Modulation Transfer Function

**DOI:** 10.3390/ma16083125

**Published:** 2023-04-15

**Authors:** Zrinka Jakopčević, Katarina Itrić Ivanda, Rahela Kulčar, Suzana Pasanec Preprotić, Marina Vukoje

**Affiliations:** Faculty of Graphic Arts, University of Zagreb, 10000 Zagreb, Croatia

**Keywords:** thermochromic ink, edge raggedness, edge blurriness, edge spread function, dot gain, print quality, modulation transfer function

## Abstract

Modern logo design is characterized by its ability to convey information through the use of various images and text compositions. These designs often use simple elements such as lines to capture the essence of a product. When using thermochromic inks in logo design, it is important to consider their composition and behavior, as they differ significantly from conventional printing inks. This study aimed to determine the resolution capabilities of the dry offset printing technique when using thermochromic ink, with the ultimate goal of optimizing the thermochromic ink printing process. Horizontal and vertical lines were printed using both thermochromic and conventional inks to compare the edge reproduction characteristics of the two ink types. Moreover, the impact of the type of applied ink on the share of mechanical dot gain of the print was investigated. Additionally, modulation transfer function (MTF) reproduction curves were generated for each print. Moreover, scanning electron microscopy (SEM) was conducted to investigate the surface of the substrate and prints. It was found that the quality of the printed edge produced by thermochromic inks can rival that of conventional inks. Thermochromic edges showed lower raggedness and blurriness values for horizontal lines, whereas line orientation proved to be insignificant in the case of vertical lines. MTF reproduction curves confirmed higher spatial resolution for vertical lines in the case of conventional inks, whereas they were identical for horizontal lines. The share of mechanical dot gain is not highly influenced by the ink type. SEM micrographs confirmed that the conventional ink smooths out the micro-roughness of the substrate. However, on the surface, the microcapsules of thermochromic ink (measuring 0.5–2 µm) are observable.

## 1. Introduction

Thermochromism refers to the phenomenon of a color change depending on the temperature. In recent decades, the field of thermochromism has made enormous progress. Thermochromic composites or pigments based on leuco dyes or cholesteric liquid crystals have become the state of the art and are described in detail [1,2,3,4,5]. The most common thermochromic inks are liquid crystals and leuco dyes used for various applications in various industries. However, the commercial applicability of liquid crystals to thermochromic products has been greatly hindered by their high cost and poor color change performance. In contrast, leuco dye systems have been much more successfully applied in a variety of commercial applications.

The dynamic change of thermochromic inks based on leuco dyes is visible when they are exposed to different temperatures. Below the activation temperature they are usually colored, and above the activation temperature they are transparent or slightly colored [6,7,8]. Very often they are mixed with some other conventional pigments in order to achieve a change from one color to another [9]. The activation temperature is defined as the temperature above which the ink has almost reached its final clear endpoint. The color starts to fade at about 3 °C below the activation temperature and will be in between the colors in the activation temperature range.

Thermochromic inks are often used as anti-forgery protection in security documents. For example, certain areas that change color after a temperature change can be created to check a document or can be protected from copying [10]. Smart sensors, systems, and indicators show the quality of packaged food products, which ensures a long shelf life, safety, and better product quality. In addition, thermochromic inks are also used in so-called smart packaging systems. They are printed on the package or label so that the consumer knows by the color of the ink whether the product is ready for consumption [11]. New ideas for applications with thermochromic inks are created every day, which is why their possibilities are being explored and improved.

All research in the area of thermochromic reproduction was conducted on full tone, especially in the area of color stability under the influence of different conditions (elevated temperature [10,11,12], exposure to UV light [13,14], and exposure to different chemicals [12,15]) and the possibilities of protecting the thermochromic ink with different coatings to prolong its properties [16,17]. There is a lack of research regarding the possibility of reproducing fine details. Preliminary investigation showed that in terms of line width reproducibility of vertical and horizontal lines with thermochromic inks, vertical lines showed lower deviations from the nominal line width regardless of the value defined in prepress (0.1, 0.5, 0.7, 1, and 1.5 pt) [18].

The primary feature of modern graphic product design is its ability to convey graphical information through various image and text compositions. Different graphical elements (typography, barcodes, logotypes, pictograms, and different designer elements) can capture and communicate the essence of a product using simple elements such as lines, enabling them to encapsulate the very essence of the product [19]. One of the ways of characterizing those elements is by their basic element definition (point, line, surface, and solid areas) [20]. Each of those basic elements has different attributes. A line is characterized by its type (straight, curve), thickness, speed, and direction (vertical, horizontal, multidirectional, and slant) [20,21]. As thermochromic inks differ significantly in composition and behavior from conventional printing inks, their reproduction on the printing substrate will not fully correspond to the predefined parameters in the preprint.

The resolution capabilities of each printing technique using thermochromic inks need to be determined in order to accurately reproduce graphic elements and to satisfy designers’ needs [22]. Therefore, it is necessary to simultaneously work on tools that will optimize the preprint, printing, and finishing processes of thermochromic ink reproduction in order to achieve a high-quality product. Graphical, structural, and verbal design cues are key elements of packaging when purchasing a product, and customers choose products based on their visual preferences [23,24]. The study of the quality of the printed element’s edge can be of great importance when working on improving and developing new color management tools. In addition, achieving high-quality print involves reproducing fine details (lines), which plays a crucial role in determining the legibility and readability of text and functionality of graphic elements [25,26,27,28,29].

Quality control of print includes parameters such as resolution and dot gain. Printers use predefined devices according to ISO standards for conventional inks, which are not suitable for special inks. It is important to note that the MTF of thermochromic ink will depend on the specific application and the requirements of the printed image. In some cases, a lower MTF may be acceptable if the thermochromic effect is the primary focus of the image. However, in other cases, a higher MTF may be necessary to ensure that the image is sharp and clear. The goal is to standardize the MTF determination method over time by developing an appropriate test form and software so that limit values for the combination of substrate and ink for each printing technique can be defined in the preparation phase.

In order to fully utilize the potential of thermochromism, it is necessary to investigate the possibility of reproducing lines of different widths and orientations, which is directly related to the readability of graphic elements printed with thermochromic inks. The thermochromic printing process, like every other, is affected by dot gain. Dot gain comprises two components: optical dot gain and mechanical dot gain. Optical dot gain is caused by the lateral scattering of light within the paper substrate resulting in the apparent raster element increase [30,31]. Mechanical dot gain is a result of the printing process. Every printing process, in all its phases, causes deformation of the raster element whether it is due to the properties of the ink and the substrate (viscosity of the ink and substrate porosity) [32,33,34] or the ink transfer followed by different drying mechanisms, while every attempt to observe the print results with the optical dot gain make the two inseparable.

Several parameters such as ESF (edge spread function), LSF (line spread function), MTF (modulation transfer function), NPS (noise power spectrum), and DQE (detective quantum efficiency) can be used to characterize the ability of an imaging system to accurately reproduce given objects. These parameters can be obtained for each detector by performing experimental measurements of a sharp edge [35,36,37,38,39,40]. Understanding the level of resolution of a system is important to comprehend how the edges of an image are blurred. Additionally, measuring the edge response is straightforward as edges can be easily generated in images. If needed, the LSF can be easily calculated by taking the first derivative of the edge response function. One of the key factors that contribute to the perceptual quality of an image is the preservation of spatial details in the imprint. In this regard, the MTF of the reproduction system determines its ability to accurately reproduce details.

Modulation transfer function is a measure of the quality of a printing system. It can give valuable information about thermochromic ink reproducibility compared with conventional methods. This paper aims to examine the possibilities of reproducing thermochromic UV-curable ink on commercially available labels in order to create a high-quality packaging product that communicates with customers. In this work, we propose a method for determining MTF curves of thermochromic line edges. In addition, edge characteristics such as edge raggedness and blurriness are examined. Moreover, the influence of printing direction is determined as well. The parameters obtained for thermochromic prints are compared with those obtained for conventional prints.

## 2. Materials and Methods

### 2.1. Materials Used in the Study

The prints were created using both UV thermochromic (TC) and conventional (C) red UV offset inks on a commercial dry offset machine designed for label printing. The prints were produced under identical conditions on commercial offset paper specifically designed for label printing. Applied thermochromic ink is blue below 29 °C, and colorless above this temperature threshold. Its viscosity is in the range of 11–18 Pa·s, which is slightly higher compared with the viscosity range of conventional UV offset ink that is typically between 2–15 Pa·s. The printing process yielded vertical lines (V) that were 1 pt wide in the machine direction, as well as horizontal lines (H) that were 1 pt wide and perpendicular to the direction of printing. Substrate characteristics are given in Table 1. The facestock is characterized with white, one-side machine-coated, wood-free printing paper with a semi-gloss appearance [41]. The mineral pigment coating formulation of the facestock is based on calcium carbonate and kaolin [42]. Roughness parameters of the substrate according to ISO 16610-21 [43] regarding arithmetical mean roughness (Ra), mean roughness depth (Rz), and maximum roughness depth (Rmax) were determined with the MarSurf PS 10 1.00-28 device.

### 2.2. Scanning Electron Microscopy (SEM)

Clear substrate surface, as well as a surface printed with both ink types, was monitored using an FE-SEM Jeol 7000 field emission scanning electron microscope (Akishima, Japan). The micrographs were taken under a magnification of 2000×.

### 2.3. Edge Quality Parameters

One of the quality parameters of the printing process is its edge reproduction. In accordance with ISO-13660 standards [44] for determination of print quality, the leading edge of a vertical line is considered to be the left edge, while the top edge is considered the leading edge of a horizontal line. Raggedness refers to the geometric distortion of an edge from its ideal position, causing it to appear rough and wavy instead of straight and smooth. It is measured as the standard deviation of the residuals from a line fitted to the edge threshold of the line under study, calculated perpendicular to the fitted line, with the edge being defined as the 60% edge threshold. Edge blurriness is another characteristic that provides valuable information regarding edge quality, as it refers to a noticeable transition of blackness from the background (paper substrate) to the real edge. Blurriness is determined as the distance between the dynamic thresholds of the 10% and 90% reflectance edge. To measure the raggedness and blurriness of the leading edges of the generated prints, the PIAS II imaging device [45,46] was employed.

### 2.4. Microscopic Imaging

The leading edge images of all printed lines were captured using the Olympus BX51 system microscope (Shinjuku, Japan) at room temperature (22 °C) to ensure the thermochromic effect of the blue ink. The Olympus BX51 microscope has the capability of capturing images under magnification up to 1500×, with program support for image adjustment and analysis. The high-resolution 12.8-megapixel (4140 × 3096 pixels) Olympus DP72 digital microscopy camera mounted perpendicular to the measuring surface enables high sensitivity (low noise) and resolution of the captured microscope samples. Ten images of every edge were captured, and the RGB data from the captured images were transformed to reflectance values using ImageJ software 1.53. The images were first converted to 32-bit gray images, and the Plot Profile tool was used to determine pixel intensity values perpendicular to the edge. The obtained intensity edge values were further processed in Origin 9.5. The average gray value of the substrate was set as 100% reflectance, while the reflectance of the ink, both conventional and thermochromic, was scaled accordingly to represent the printed ink reflectance value. Localized spikes in the reflectance profiles (noise) were smoothed using median filtering with a 10-point window. The removal of noise is necessary due to its influence on the derivation of the reflectance profile. During differentiation, generated noise is even greater than the one from the reflectance profile, preventing the approximation of the measured profiles by analytical functions.

### 2.5. Procedure of Generating MTF

MTFs were generated according to the procedure previously published by Itrić et al. [47]. Reflectance spectra were further processed in Origin 9.5. software. Since the edge profile is influenced by both optical and mechanical dot gain, which are governed by different physical processes, the spread function is not symmetric [40]. Consequently, the resulting edge profiles were fitted with bi-logistic growth function according to Equation (1):

(1) $$f=a_{1}+\left(a_{2}-a_{1}\right)\cdot \left[\frac{p}{1+10^{\left(b_{1}-x\right)h_{1}}}+\frac{1-p}{1+10^{\left(b_{2}-x\right)h_{2}}}\right]$$

The minimum and maximum reflectance values are denoted by *a*_1_ and *a*_2_, respectively. The inflection points are represented by *b*_1_ and *b*_2_. The slopes *h*_1_ and *h*_2_ correspond to the mechanical/optical dot gain, while *p* represents the fraction of the phase with the greater contribution ratio. *LSF* is the derivative of the fitted bi-logistic growth function according to Equation (2):

(2) $$LSF\left(x\right)=\frac{d\left[ESF\left(x\right)\right]}{dx}$$

*MTFs* from obtained *LSFs* were calculated using built-in *FFT* function in Origin 9.5. *MTF* was normalized to unity at zero spatial frequency according to Equation (3):

(3) MTF(ω)=FFT|{LSF(x)}|

## 3. Results and Discussion

**Figure 1:**
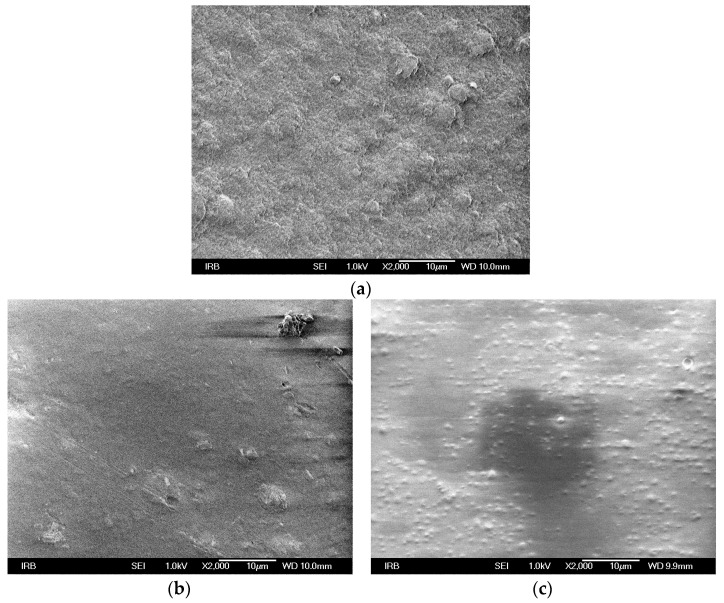
SEM micrographs of (**a**) unprinted substrate; (**b**) conventional ink; and (**c**) thermochromic ink.

SEM micrographs of the unprinted substrate, as well as those printed with conventional and thermochromic ink are given in Figure 1. The application of conventional ink onto a coating (Figure 1b) results in the coverage of micro-roughness features present on paper (Figure 1a). The micro-roughness features comprise small crevices and valleys on the surface of the paper, which become filled with ink, producing an even layer of ink on top, thereby rendering a smooth finish. Pigment particles of conventional ink cannot be observed. In the case of thermochromic ink (Figure 1c), microcapsules can be observed on the surface which indicates that the ink has remained on the surface of the paper. The size of the capsules ranges from 0.5 µm to 2 µm.

**Figure 2:**
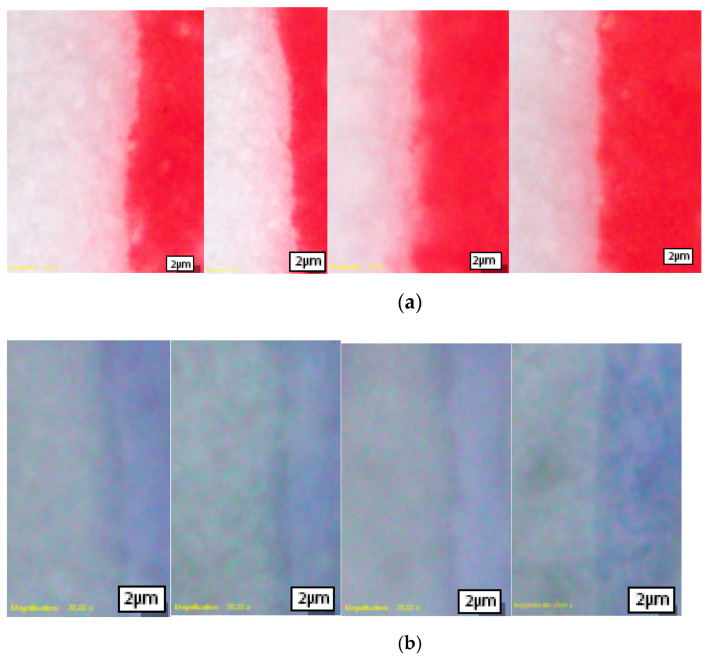
Vertical edge profiles: (**a**) conventional ink; (**b**) thermochromic ink.

**Figure 3:**
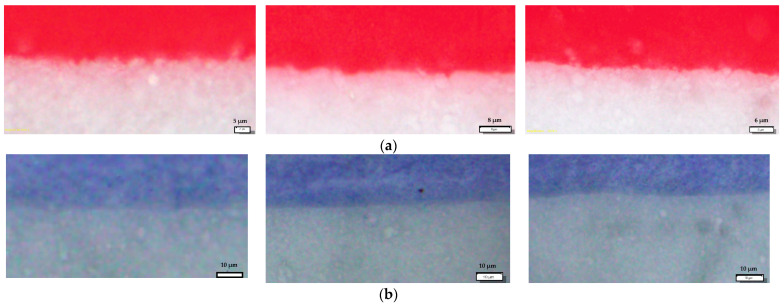
Horizontal edge profiles: (**a**) conventional ink; (**b**) thermochromic ink

The unprocessed microscopic images of all samples are presented in Figure 1. It is apparent that the conventional red ink (Figure 2a and Figure 3a) provides greater color coverage with the same amount of applied ink, while on the TC prints (Figure 2b and Figure 3b), the paper substrate is visible breaking through the print.

Leading edge blurriness varies significantly for horizontal and vertical edges regardless of the applied ink (Table 2), which is caused by the substrate structure. Paper, as a cellulose-based substrate, exhibits both optical and mechanical imperfections. The main reason lies in the heterogeneity of the fibrous material, starting from the topography of the surface and ending with local variations in tone. Moreover, it can be observed that the blurring of the edge of the vertical lines is almost the same for both types of applied ink. Regarding horizontal edges, significant difference in blurriness can be observed. Horizontal edges generated with conventional ink show a seven times higher level of blurriness in comparison with horizontal edges generated with thermochromic ink. As edge blurriness is directly linked with reproduction quality of high-frequency details, its values imply that fine details can be better reproduced with thermochromic ink; hence, there are no obstacles in the design and printing of complex logotypes or other design cues.

When ink is printed on paper, it tends to spread more in the direction of the paper fibers than across them. This means that if a dot is printed parallel to the paper fibers, it will spread out more in that direction, resulting in a larger printed dot than intended. This effect is known as fiber elongation. Conversely, if a dot is printed perpendicular to the paper fibers, it will spread out less in that direction, resulting in a smaller printed dot than intended. This effect is known as fiber compression [48]. Moreover, since the applied thermochromic ink is more viscous, it spreads less, resulting in a more accurate print.

There can be a connection between line blurriness and ink viscosity. If the ink viscosity is too low, it can cause the ink to spread more than desired, resulting in blurred lines. On the other hand, if the ink viscosity is too high, it can result in poor ink transfer and uneven coverage, which can also affect line clarity. Therefore, finding the optimal viscosity of ink is crucial for achieving sharp and clear lines. Other factors, such as paper quality, printing speed, and pressure, can also affect line blurriness.

Moreover, line raggedness and ink viscosity can be linked. Ink viscosity refers to the thickness or fluidity of the ink. When ink viscosity is too high, it can cause the ink to not flow smoothly onto the paper substrate, resulting in irregularities or bumps in the printed line. This can lead to raggedness or waviness in the printed line, which is a measure of geometric distortion from its ideal position. On the other hand, if the ink viscosity is too low, it can cause the ink to spread too much over the paper substrate, resulting in a blurry or fuzzy printed line. Therefore, finding the appropriate ink viscosity is crucial in obtaining smooth and sharp printed lines without raggedness or blurriness. The sizes of the microcapsules in offset TC inks are not uniform; they vary from 1–5 µm [49]. Previous research showed that TC microcapsules penetrate within the paper substrate in accordance with their size. Smaller microcapsules thus penetrate deeper inside the paper structure while larger ones stay on the paper surface [49]. In the case of the applied UV thermochromic offset ink, the microcapsules are significantly smaller 0.5–2 µm (Figure 1c). The amount of photoinitiator in UV ink formulation can vary from 0.5 to 15 %. The formation of necessary radicals for curing of ink vehicle can be impeded by the presence of colored pigments, leading to reduced light accessibility of the photoinitiator. This makes the process of colored layers more difficult to cure in comparison with white coatings. The ability of the pigment to hide is determined by the scattering coefficient, which is dependent on the particle size of the pigments and the difference in refractive indices between the pigment and the vehicle. To estimate the efficiency of UV-curing, the integrated transmission spectrum, which includes scattering and reflection contributions in the critical wavelength region where the UV-initiators absorb and form radicals to initiate the polymerization reaction, can be used [50,51,52].

Leading edge raggedness varies significantly within the samples (Table 2). Since edge raggedness is in correlation with the edge roughness, it can be concluded that horizontal thermochromic edges show the best quality. During the dry offset printing process, vertical lines are rougher due to the roller rotation being parallel to the printing edge direction. Moreover, conventional edges show a higher level of raggedness which can be explained with the slower drying process. Although both inks are UV curing, photo initiators in thermochromic ink are more efficient causing faster polymerization and thus are less prone to smearing. Larger pigment particles have a smaller surface area relative to their volume, which means they are less efficient at absorbing and scattering UV radiation. This can improve the efficiency of UV curing by allowing more UV radiation to reach the photoinitiator molecules and initiate the polymerization reaction more efficiently [50,51,52].

**Figure 4:**
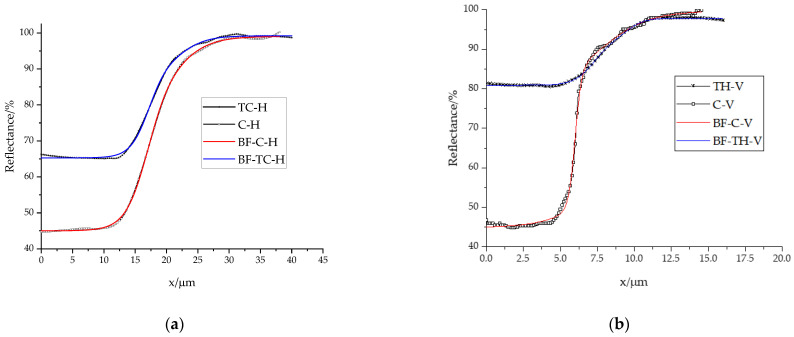
Measured and bi-dose-fitted (BF) reflectance profiles of conventional (C) and thermochromic (TC) prints: (**a**) horizontal edge profiles; (**b**) vertical edge profiles.

The edge spread function obtained by the Bi-logistic function fitting of all four printing ink and direction combinations corresponds to the measured edge profiles (Figure 4). From Figure 4a depicting horizontal edges, it can be seen that the reflectance spectra of the ink layer (0–12.5 µm) are 20% lower for conventional ink than for thermochromic ink due to the better ink coverage of conventional ink which result in higher absorbance. Previous research has shown that inks with higher viscosity produce higher ink density values when discussing conventional inks [53]. Fine particles produce a uniform and glossy surface because the angle of incident light is close to the angle of reflected light. In this context, pigments in conventional inks can be considered as fine particles compared with the size of pigments in thermochromic inks. Aside from the hiding power, particle size has an influence on the viscosity of pigment in a vehicle. There is an increase in viscosity when finer particles are used [52]. Paper influences and increases the overall reflectance. Around 27.5 µm, the reflectance of both prints is almost equal, at around 100 %, and this reflectance corresponds to substrate reflectance. As for vertical edges (Figure 4b), these differences in the reflectance of the area covered in ink are even more pronounced. Conventional ink shows a similar reflectance value of 45%, while the reflectance of the thermochromic ink layer is higher than 80%. It is evident that the printing direction significantly influences the reproducibility of the edge and its characteristics.

However, in general, thermochromic inks tend to have lower surface coverage than conventional inks due to their unique properties, i.e., smaller concentration of TC pigments in comparison with conventional pigment due to their larger particle size. Thermochromic inks contain microencapsulated pigments dispersed in ink vehicle that change color in response to temperature, which can result in a thinner ink film on the substrate surface.

Table 3 gives the parametric values of bi-logistic fit function arguments for all examined edges. The maximum reflectance value (*a*_2_) corresponding to paper reflectance is from 97 to almost 100%, while the minimum values for conventional printed edges show identical values.

In our previous research that examined the ratio of mechanical dot gain in the overall dot gain of electrophotographic prints with dry toner, it was observed that mechanical dot gain is the more dominant phase with a share of 70–75%, while the remaining part corresponds to optical dot gain [47]. Similar results can be confirmed in the case of both thermochromic and conventional dry offset prints for horizontal edges (75–77%). Considering that the paper substrate is coated, the degree of optical dot gain conditioned by its optical and mechanical properties is lower. Moreover, as in the case of electrophotographic prints [47], a decrease in the share of mechanical dot gain can be observed with vertical lines, regardless of the type of the ink, although the phenomenon is more pronounced with conventional inks (64% mechanical dot gain).

**Figure 5:**
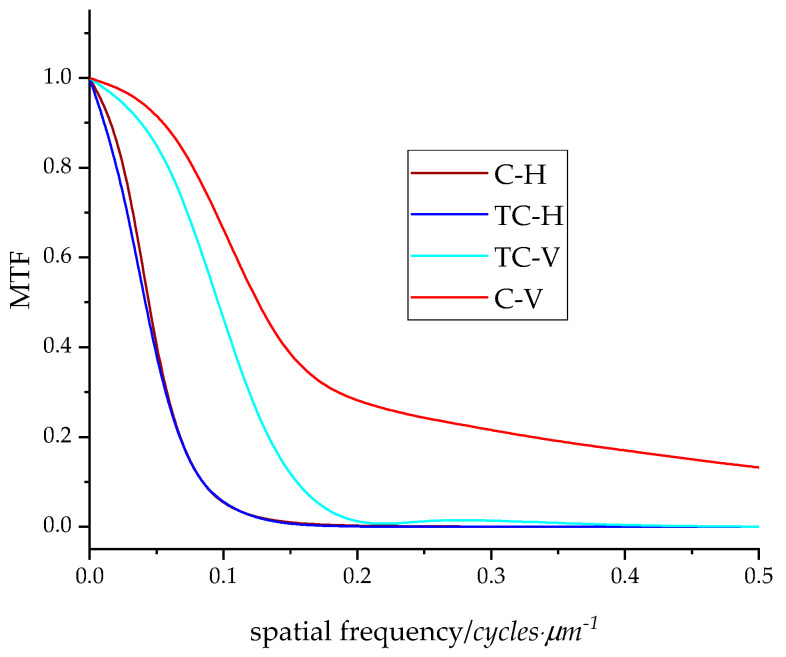
MTF profiles of thermochromic horizontal edge (TC-H), conventional horizontal edge (C-H), thermochromic vertical edge (TC-V), and conventional vertical edge (C-V).

The modulation transfer function reproduction curves are given in Figure 5. As in previous studies examining the influence of print direction on the width of the MTF reproduction curve, it is observed that the reproduction of information is better for vertical lines [54]. Moreover, MTF reproduction curves for horizontal edges almost match for both ink types. For high frequencies, reproduction is of the highest quality for vertical edges printed with conventional UV ink. This can be explained by the sizes of TC microcapsules and conventional ink pigment. Conventional ink pigment is ten times smaller than TC microcapsules. Furthermore, the distinction between the printing edge direction MTFs is more pronounced for conventional inks. The spatial frequency at 50% modulation should provide an estimate of the perceived sharpness of edge detail; the spatial frequency at 10% modulation provides an estimate of the limiting resolution of edge detail [55]. Even though the MTF curves for horizontal print edges are almost identical, the data of the MTF value at 50% (Table 4) indicate a higher spatial frequency resolution for conventional horizontal prints. As for vertical edges, the spatial frequency at 50% MTF are significantly higher for conventional ink prints, indicating higher resolution and better image quality.

The 10% MTF value is important because it indicates the spatial frequency limit of the imaging system. If an imaging system has a low 10% MTF value, it means that the system has poor spatial resolution and cannot resolve fine details in the object. On the other hand, an imaging system with a high 10% MTF value can capture fine details in the object and produce high-quality images. The results indicate that vertical lines have higher spatial resolution than horizontal ones. Moreover, the ink’s effect on horizontal print edges does not depend on the applied ink but is a result of the printing process and substrate. Whereas for vertical lines, the effect of the applied ink is evident.

## 4. Conclusions

The limited availability of research on the quality of line reproduction using thermochromic inks hinders their wider use in the design of basic graphic elements such as lines. Today’s consumers are highly visually oriented when selecting products, so the possibility of using thermochromic inks in logo design on labels and packaging is one way to make a product stand out. Additionally, from a sustainability and environmental perspective, it is desirable to use as little ink as possible and avoid heavy ink coverage when possible. Due to their high cost compared with commercial inks, it is necessary to fully optimize the printing process with thermochromic inks, whether it be color management, edge quality, or resolution capabilities. With this goal in mind, horizontal and vertical edge profiles were generated with both thermochromic UV-curable and conventional UV-curable ink.

The SEM micrographs confirmed that the used substrate is coated; therefore, penetration of the ink in the substrate can be neglected. Moreover, conventional ink printing results in a smooth finish by covering the micro-roughness features of the substrate, while thermochromic ink prints reveal the presence of microcapsules on the paper surface which vary from 0.5 to 2 µm. The study confirmed that in terms of edge reproduction quality, thermochromic UV-curable inks are competitive with conventional ones. When comparing the leading edge raggedness of conventional and thermochromic UV-curable inks, it is observed that raggedness is higher for conventional inks. Additionally, the vertical edges of both prints have similar values, while the values of raggedness for horizontal lines are significantly different and significantly higher for conventional inks. Leading edge blurriness shows almost identical values for vertical edge profiles for both inks, while blurriness is especially high for the horizontal edge with conventional ink. A comparison of MTF reproduction curves gave valuable information regarding thermochromic print sharpness and resolution properties. Vertical lines have higher spatial resolution than horizontal lines, and the ink’s impact on horizontal print edges is a result of the printing process and substrate, rather than the applied ink. However, the type of applied ink does have an effect on vertical lines. The MTF curves of horizontal print edges show almost identical results, but the MTF value at 50% indicates higher spatial frequency resolution for conventional horizontal prints. In contrast, vertical edges show significantly higher spatial frequency at 50% MTF for conventional ink prints, indicating better image quality and higher resolution. The share of the mechanical dot gain is not highly influenced by the ink type.

In future research, the impact of the substrate on the quality of edge reproduction will be investigated to further obtain relevant data which will serve as a reference for color management software and print optimization.

## Figures and Tables

**Table 1:** Substrate characteristics.

Sample	Opacity	Brightness/ISO	Lightness	Ra/µm	Rz/µm	Rmax/µm
	100 ± 0	76.5 ± 0.5	80 ± 2	0.566	3.455	4.493

**Table 2:** Average edge raggedness and blurriness values for the thermochromic horizontal edge (TC-H), conventional horizontal edge (C-H), thermochromic vertical edge (TC-V), and conventional vertical edge (C-V).

Sample	Raggedness/µm	Blurriness/µm
TC-H	4.0 ± 0.3	101 ± 9
C-H	24 ± 3	734 ± 46
TC-V	13 ± 2	430 ± 10
C-V	17.0 ± 0.5	451 ± 45

**Table 3:** Parametric values of bi-logistic fit function arguments for thermochromic horizontal edge (TC-H), conventional horizontal edge (C-H), thermochromic vertical edge (TC-V), and conventional vertical edge (C-V).

Sample	*a*_1_/%	*a*_2_/%	*b*_1_/µm	*b*_2_/µm	*h*_1_/µm^−1^	*h*_2_/µm^−1^	*p*
TC-H	65.28 ± 0.06	99.21 ± 0.08	17.2 ± 0.1	23 ± 1	0.29 ± 0.01	0.20 ± 0.03	0.77 ± 0.07
C-H	44.99 ± 0.07	99.0 ± 0,1	16.9 ± 0.2	22 ± 2	0.25 ± 0.01	0.15 ± 0.02	0.75 ± 0.11
TC-V	80.88 ± 0.05	97.89 ± 0.04	7.3 ± 0.1	9.5 ± 0.2	0.64 ± 0.04	0.72 ± 0.09	0.68 ± 0.06
C-V	44.9 ± 0.2	99.7 ± 0.2	5.99 ± 0.01	7.7 ± 0.1	1.96 ± 0.09	0.27 ± 0.01	0.64 ± 0.01

**Table 4:** Spatial frequencies at 50% MTF values and 10% MTF values for thermochromic horizontal edge (TC-H), conventional horizontal edge (C-H), thermochromic vertical edge (TC-V), and conventional vertical edge (C-V).

Sample	Spatial Frequency at 50% MTF/Cycles·µm^−1^	Spatial Frequency at 10% MTF/Cycles·µm^−1^
TC-H	0.041495	0.083561
C-H	0.044459	0.083561
TC-V	0.094847	0.153215
C-V	0.125969	0.605107

## Data Availability

The data presented in this study are available in article.
